# A 57-year-old Brazilian woman with a giant mucinous cystadenocarcinoma of the ovary: a case report

**DOI:** 10.1186/1752-1947-8-82

**Published:** 2014-03-04

**Authors:** Sergio Henrique Mattioda de Lima, Vitorino Modesto dos Santos, Andersen Charles Darós, Victor Paranaíba Campos, Fabiana Ruas Domingues Modesto

**Affiliations:** 1 Gynecology and Obstetrics Division of Armed Forces Hospital (HFA) and South Wing Regional Hospital (HRAS), Brasília-DF, Brazil; 2 Internal Medicine Department of HFA, and Catholic University of Brasília (UCB), Brasília-DF, Brazil; 3 Pathology Division of HFA, and HRAS, Brasília-DF, Brazil; 4 Gynecology and Obstetrics Division of Brasilia University Hospital (HUB), Brasília-DF, Brazil; 5 Brazilian Federation of Gynecology and Obstetrics (Febrasgo), São Paulo-SP, Brazil; 6 Department of Medicine, Armed Forces Hospital (HFA), Estrada do Contorno do Bosque s/n, Cruzeiro Novo, 70630-900 Brasília-DF, Brazil

**Keywords:** Giant, Cystadenocarcinoma, Mucinous, Ovary, Tumor

## Abstract

**Introduction:**

Giant cystadenocarcinomas of the ovary are rarely described conditions.

**Case presentation:**

The authors describe a 57-year-old Brazilian woman who presented with an increase in abdominal girth in February 2003. Imaging studies showed a giant abdominal pelvic mass with probable origin in the right ovary. Cancer antigen-125 was elevated, while carcinoembrionic antigen and alpha-fetoprotein were normal. Total abdominal hysterectomy, bilateral salpingoophorectomy and omentectomy were done. The mass weighed 40Kg, and the histopathology study revealed a mucinous cystadenocarcinoma. She underwent chemotherapy with paclitaxel and cisplatin with no side effects. Under follow-up for more than 10 years, she is asymptomatic and with normal imaging and laboratory parameters, including the cancer antigen-125 marker.

**Conclusion:**

This huge tumor evolved for a long time unsuspected and without metastases in a patient from a developing region. The diagnostic and management challenges posed by this unexpected and unusual presentation of an ovarian cystadenocarcinoma are discussed.

## Introduction

Mucinous cystadenomas have origins from inclusions and invaginations of the ovarian celomic epithelium and persistence of Müllerian cells, or from Wolffian epithelium and teratomas [1]. They often occur in the fourth and fifth decades, accounting for 25% of the ovarian tumors, 5% are bilateral and 15% are malignant [1]. Mucinous (25%) and serous (75%) cystadenomas account for 8 to 15% of all ovarian tumors [2,3]. The epithelium of the cysts is usually cylindrical and mono- or multi-stratified, and cuboidal epithelium is due to the pressure inside the cyst [1]. The classical cells show clear cytoplasm and a hyperchromatic nucleus at the base [1].

Extra-large benign and malignant cysts of the ovary are uncommon and involve diagnostic and management challenges [2-7], and determinations of cancer antigen (CA)-125 can help to identify epithelial tumors of the ovary [2]. Giant mucinous cystadenocarcinomas are very rare [3,7].

This report concerns an unsuspected giant mucinous ovarian cystadenocarcinoma in a 57-year-old woman with a huge abdominal enlargement. The main objective of this report is to call attention to ovarian epithelial cysts in the outpatient clinics and primary care services, contributing to a decrease in any under-diagnosis, misdiagnosis and underreporting that might occur.

## Case presentation

**Figure 1:**
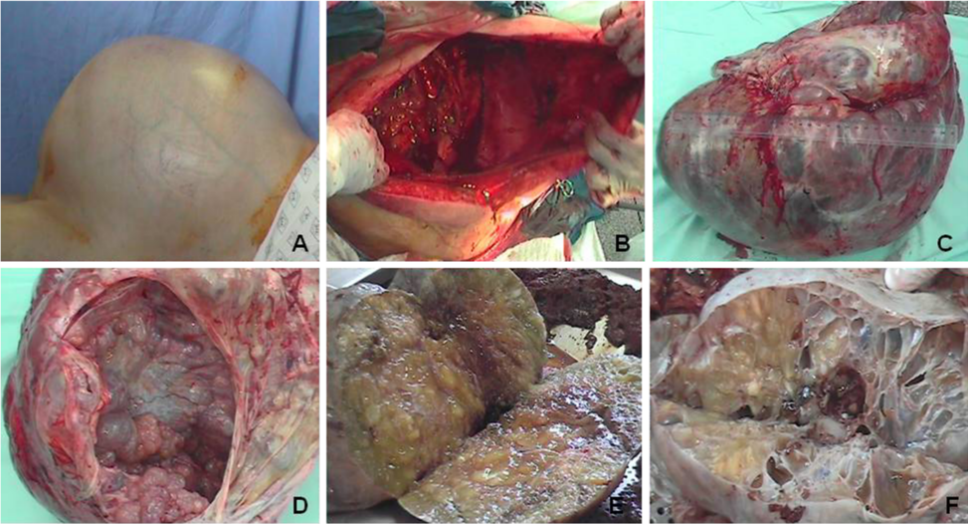
**Gross features of the giant mucinous cystadenocarcinoma of the ovary. (A)** Preoperative aspect of the massively enlarged abdomen with superficial venous congestion due to compressive effects of the ovarian mass.** (B)** Accentuated excess of skin and subcutaneous tissue observed after removal of the huge tumor. **(C)** Gross view of the bulky, multicystic tumor with a smooth external surface and vascular congestion. **(D)** The internal surface shows multiple rounded projections. **(E and F)** The sectioned surface reveals large multiloculated cysts containing watery and viscous mucoid material.

**Figure 2:**
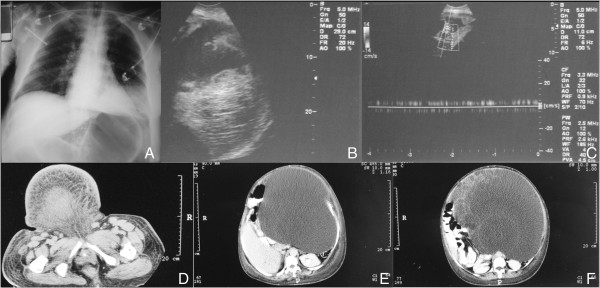
**Imaging studies of the giant mucinous cystadenocarcinoma of the ovary. (A)** Chest radiograph showing an upward compression of the diaphragm. **(B)** Ultrasound images of the heterogeneous mass, which occupies the entire abdominal cavity. **(C)** Abdominal ultrasound (US) showing normal liver, spleen and kidneys. **(D-F)** Contrasted computed tomography (CT) scan revealing an extra-large mass with well-defined limits, and heterogeneous coefficient of attenuation, which was predominantly liquid. The mass appeared to extend from the right adnexal area up to the diaphragmatic region, displacing the abdominal contents.

**Figure 3:**
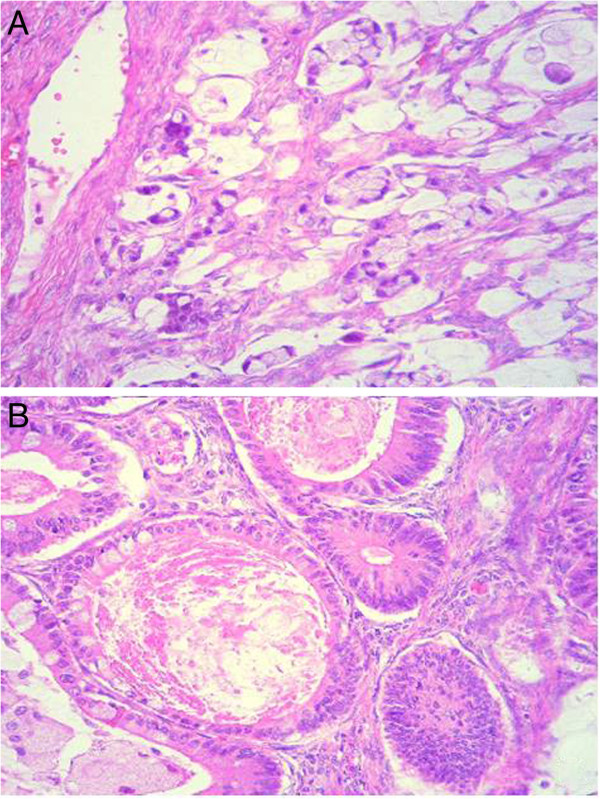
**Photomicrograph of the ovarian mucinous adenocarcinoma with infiltrative and expansile growth patterns. (A)** The irregular glands are lined by cells with malignant features, which infiltrate the stroma and present the characteristic abundant cytoplasm (hematoxylin and eosin×40). **(B)** The expansile invasive pattern of the tumor glands, which are lined by cells with atypical nuclei and some intracytoplasmic mucin vacuoles (hematoxylin and eosin × 40).

A 57-year-old Brazilian woman was referred to our hospital because of an increase in abdominal girth in February 2003. She said she was overweight, and had noted a rapid increase in abdominal girth during the last year. Her menarche occurred at the age of 15, she always had regular periods, and had used oral contraceptives for two years. She had eight pregnancies, producing eight children, and underwent tubal ligation at the age of 29. Her menopause occurred at the age of 46, and she never used hormonal therapy. She underwent an appendectomy and cholecystectomy, respectively, at the ages of 16 and 29, and had arterial hypertension and dyslipidemia. She denied the use of tobacco, alcohol or illicit drugs. Physical examination revealed pale skin, a temperature of 36.1°C (97°F), a respiratory rate of 25 breaths/minute, a pulse rate of 100 beats per minute, blood pressure at 180/140mmHg; normal cardiac and respiratory sounds; her abdomen was distended and tense, and her abdominal circumference measured 134cm (Figure 1A), with dullness to percussion and superficial dilated veins. Examination of her breasts, vulva and vagina did not reveal abnormalities. Her lower limbs showed moderate swelling. She was admitted to the gynecological ward for accurate diagnosis and treatment. Laboratory tests revealed hematocrit 29%, hemoglobin 9.2g/dL, leukocytes 7000 cells/μL, platelets 478,000/μL, CA-125 374.8IU/dL, and normal levels of carcinoembrionic antigen (CEA) as well as of alpha fetal protein. The chest radiograph showed an upward compression of her diaphragm, and abdominal ultrasonography study showed a voluminous cystic abdominal-pelvic mass with thick walls, strongly suggestive of an ovarian tumor (Figures 2A, B). Abdominal ultrasound (US) images showed a normal aspect of the liver, bile ducts, portal and supra-hepatic veins, as well as normal spleen and kidneys. The cystic mass had rough septa and voluminous solid components, occupying the entire abdominal cavity with extrinsic compression of the liver and spleen; discrete blood flux within the tumor and high resistance peripheral blood flow. Computed tomography (CT) images of the abdomen and pelvis revealed a conspicuous and well delineated tumor with heterogeneous attenuation coefficient, predominantly liquid and with coarse septa and some solid internal irregularities. The huge tumor displaced her uterus to the left, compressed her abdominal contents, and extended up to her diaphragm (Figures 2D-F). As the main hypothesis was a giant adnexal complex mass, in addition to the high possibility of malignant origin, the surgical option was open laparotomy. Procedures included the intact removal of the right ovarian tumor along with her normal right fallopian tube, left adnexal resection, total hysterectomy, omentectomy, para aortic and pelvic lymph node dissection to stage the disease, and sampling of lymph nodes and of the ascitic fluid for routine tests and cytopathological evaluations. Accentuated excess of skin and subcutaneous tissue was observed after removal of the mass (Figure 1B). The extra-large (42×40×28cm) tumor, weighing 40kg, had a smooth external surface, and the inner aspect was multilocular, with mucinous and serous fluid areas (Figures 1C-F). The histopathological diagnosis was a well-differentiated mucinous cystadenocarcinoma (Figure 3). The staging was 1A (tumor limited to one ovary, no ascites present containing malignant cells, no tumor on the external surface, and capsule intact). The immediate postoperative period was in the Intensive Care Unit (ICU), for close hemodynamic, ventilatory and renal function monitoring and fluid and electrolytes control. She underwent six cycles of chemotherapy (three weeks apart) with paclitaxel plus cisplatin, with no side effects, and was under close surveillance on the outpatient section of Clinical Oncology. The option for intravenous chemotherapy in this setting was based on two major concerns. The very large abdominal cavity made it difficult to treat with intraperitoneal chemotherapy with platin, and the persistence of elevated CA-129 after the cytoreduction. The levels of CA-125 were 374.8 and 222IU/mL (before and after the cytoreductive surgery), and post chemotherapy control was 7.1IU/mL. Actually, carboplatin is the best option because of its less toxic effects; however, cisplatin was the standardized drug in our hospital at the time of her admission. She has been under specialized follow-up for more than 10 years, asymptomatic and with normal imaging and laboratory parameters, including the CA-125 marker.

## Discussion

Ovarian cysts are considered large if they have diameters between 5 and 15cm [3,8] or between 10 and 20cm [9]; and those with diameters over the respective upper limits are called giant cysts. The largest cystic tumor of the ovary weighed 137.4kg and was removed intact by O’Hanlan in 1994 [6]. Another giant (64kg) mucinous tumor with foci of a well-differentiated adenocarcinoma was described by Poole *et al.* in a morbidly obese woman [7]. In young people, the majority of ovarian cysts decrease in size or even disappear and therefore should be dealt with with a careful expectant follow-up by ultrasonography. Benign cysts of less than 8cm are conservatively managed, but cystectomy is indicated for cysts over 5cm in post-menopausal women. Giant cysts require resection because of compressive symptoms or risk of malignancy and their management invariably requires laparotomy to prevent perforation and spillage of the cyst fluid into the cavity [8]. Clinically, the differential diagnosis of large abdominal masses should include: uterine enlargement (pregnancy, fibromyomatosis); pelvic endometriosis; (pregnancy, abdominal cysts); abdominal pregnancy; urinary retention (full bladder); intestinal tumors; hydronephrotic kidney; pelvic retroperitoneal tumors; accentuated obesity; ascitis; cyst of the urachus; mesenteric cyst; abdominal cocoon [10]; and echinococcosis [11], among others [1,2,8].

Women with abdominal-pelvic masses constitute a challenging condition in general practice, because the clinical features and findings from physical examination are usually nonspecific. Moreover, concomitance with overweight and obesity can be additional diagnostic pitfalls. Imaging studies of the abdomen can contribute in ruling out the main alternative hypotheses. Although tumor markers can be a useful tool for differential diagnosis of malignant cysts, some authors have described elevated levels of these markers in patients with benign tumors. Cevik and Guldur reported a 13-year-old patient with abdominal enlargement and pain due to a giant (40×30×20cm) mucinous cystadenoma of the left ovary. The levels of CEA, CA 19–9 and CA-125 were high, but α-fetoprotein and human chorionic antigen were normal [2]. Gorgone *et al.* described a 17-year-old patient presenting with abdominal pain, vomiting and fever, associated with a giant (20×14×6.5cm) mucinous cystadenoma of the right ovary [1]. Jones *et al.* removed by laparotomy a giant (21kg) mucinous cystadenoma of the right ovary detected in a 52-year-old patient who presented with morbid obesity and acute abdominal pain [3]. Willems *et al.* reported two 15-year-old girls with giant benign ovarian tumors (teratoma and cystadenoma) presenting with different clinical features and elevation of tumor markers [12]. A giant (30kg) mucinous cystadenoma was removed by Zanini *et al.* utilizing open surgery in a 55-year-old patient who presented with intense abdominal pain soon after a paracentesis to rule out the hypothesis of liver cirrhosis. Determinations of tumor markers were normal [13].

Major diagnostic difficulties are often posed if inner nodules are disclosed in these cystic cavities, because this finding must be considered as indicative of a malignant tumor [14,15]. In our present case study, the giant ovarian tumor was multilocular with diverse inner solid masses, and the histopathology evaluation characterized the diagnosis of cystadenocarcinoma. Chakrabarti *et al.* described two cases of benign mural nodules suggesting sarcomas in right ovarian mucinous cystadenomas; one of them was a giant (22×18cm) mass. In both cases, the women described a progressive infraumbilical swelling and abdominal pain [14]. Malignant tumors (anaplastic carcinoma, carcinosarcoma, fibrosarcoma, rhabdomyosarcoma, undifferentiated sarcoma), mixed nodules and leiomyoma among others, were ruled out [14].

Based on cell origin [15], ovarian tumors are classified as germ-cell tumors (undifferentiated and extra-embrionic); stromal tumors (granulosa-theca and Sertoli or Leydig cells); and epithelial tumors (cystadenoma, borderline cystadenoma (CAdB), and cystadenocarcinoma). Heinen *et al.* described a 13-year-old girl with an asymptomatic giant (25×25cm) CAdB on the left ovary. The tumor marker evaluation was negative, and a Pfannenstiel laparotomy was performed [15]. The interval between the symptoms’ onset and clinical presentation of ovarian cancer is a major concern about diagnostic challenges involving this malignancy. Ovarian tumors are included among malignancies with shorter symptom-to-visit interval. Nevertheless, symptoms frequently develop insidiously and with intervals usually longer in the localized disease [16]. Noteworthy is that delay in ovarian cancer detection has a direct relationship with poor outcome [16], but some examples of longstanding localized evolution have been reported.

Our patient presented with an asymptomatic increase in abdominal girth, which was associated with a giant mucinous cystadenocarcinoma found in her right ovary. She did not search for medical help for one year of the abdominal change, which allegedly was self-interpreted as an increase in her usual overweight.

## Conclusion

Despite diagnostic delay, the cystadenocarcinoma was removed before any dissemination. Our present case study gives evidence of unsuspected development of a malignant giant abdominal tumor in a woman from a developing region. Case reports contribute to an increase in the suspicion index about uncommon conditions in primary care settings and in specialized services, and may decrease possible under-diagnosis, misdiagnosis and underreporting.

## Consent

Written informed consent was obtained from the patient for publication of this case report and accompanying images. A copy of the written consent is available for review by the Editor-in-Chief of this journal.

## Abbreviations

Abdominal US: Abdominal ultrasonography; CA-125: Cancer Antigen-125; CAdB: Borderline cystadenoma; CEA: Carcinoembrionic antigen; CT: Computed tomography.

## Competing interests

The authors declare that they have no competing interests.

## Authors’ contributions

SHML and VMS were the major contributors in writing the manuscript, and interpreted the clinical, complementary and surgical data. ACD performed the macroscopic evaluation of the tumor and interpreted the histological examinations. SHML and VPC participated in the ovarian tumor operation and treated the patient. FRDM was a contributor in writing the manuscript, and did the literature search. All authors read and approved the final manuscript.
